# Long-term microclimate study of a peatland in Central Europe to understand microrefugia

**DOI:** 10.1007/s00484-022-02240-2

**Published:** 2022-02-03

**Authors:** Sandra Słowińska, Michał Słowiński, Katarzyna Marcisz, Mariusz Lamentowicz

**Affiliations:** 1 Climate Impacts Laboratory, Department of Geoecology and Climatology, Institute of Geography and Spatial Organization, Polish Academy of Sciences, Warsaw, Poland; 2 Past Landscape Dynamics Laboratory, Institute of Geography and Spatial Organization, Polish Academy of Sciences, Warsaw, Poland; 3 Climate Change Ecology Research Unit, Faculty of Geographical and Geological Sciences, Adam Mickiewicz University, Poznań, Poland

**Keywords:** Microclimate, Peatlands, Local climate, Climate change refugia, Air temperature, Vapor pressure deficit

## Abstract

Peatlands perform many important ecosystem functions at both the local and global scale, including hydrologic and climatic regulation. Although peatlands often act as climatic microrefugia, they have rarely been the subject of long-term microclimatic studies. In this study, we aimed to compare the local climatic conditions of a mid-forest mire to that of an open area and examine the differences in microclimates within the mire based on plant community diversity, shading, and water table depths. The peatland studied in this work was significantly cooler than the reference site, mainly due to a higher decline in nighttime air temperatures. However, the daily maximum air temperature near the ground was often higher. We also noticed that microclimates significantly differed within the studied peatland. Wet and shaded microsites were cooler than the sites having a lower water level and receiving higher amounts of solar radiation. The results of the study suggest that peatlands have locally cooler climates, and thus can serve as climate change refugia. These findings can help us interpret reconstructed data from the peat archive, and, when combined with experiments, identify tipping points for peatland ecosystems.

**Supplementary Information:**

The online version contains supplementary material available at 10.1007/s00484-022-02240-2.

## Introduction

Peatlands, like any other ecosystem, are affected by climate change, which is expected to worsen in the coming decades (Malhi et al. 2020). Climate change causes a variety of disturbances in peatlands, ranging from hydrological to trophic changes, which are further exacerbated by detrimental human land-use practices. Although peatlands cover only 3% of the earth’s surface, they serve as carbon sinks, water reservoirs, habitat for specific species, and natural paleoenvironmental archives (Amesbury et al. 2019; Jassey et al. 2018; Nichols and Peteet 2019). Undisturbed peatlands (mires) act as net carbon sinks and contribute to global cooling if water inflow (precipitation, groundwater supply) exceeds water losses (evapotranspiration, runoff), and thus become the habitats of special management and protection (Limpens et al. 2008; Moore et al. 2002). Some ecosystems quickly show the effects of climate change than others (Bertrand et al. 2011; Settele et al. 2014; von Arx et al. 2012). Therefore, researchers have been performing intensive experimental studies on the response of peatland ecosystems to climate changes (Buttler et al. 2015; Delarue et al. 2011; Dieleman et al. 2015; Reczuga et al. 2020; Turetsky et al. 2011).

Despite often acting as microrefugia, as evidenced by the results of palaeoecological research, peatlands have rarely been the subject of long-term microclimatic studies (Dítě et al. 2018; Jones et al. 2009; Wieder 2006). Microrefugia are sites displaying favorable local conditions that allow species populations to survive beyond their main distributions during unfavorable regional climatic conditions (Dobrowski 2011). Morelli et al. (2016) defined climate change refugia as areas relatively buffered from contemporary climate change over time that enable persistence of valued physical, ecological, and socio-cultural resources, and highlighted their importance as a climate change adaptation tool.

The existence of a given refugium may be determined by external factors, such as terrain location (terrain-mediated refugia) (Dobrowski 2011, Straberg et al. 2020) and water availability (hydrological refugia) (McLaughlin et al. 2017), as well as internal regulatory processes (ecosystem-protected refugia) (Shur and Jorgenson 2007; Waddington et al. 2015). Usually, several factors work together in synergy. According to paleoecological studies, some peatlands can resist disturbances and remain resilient for thousands of years (Lamentowicz et al. 2019a; Łuców et al. 2020).

Climate change forces living organisms to adapt to local climates or become extinct (Birks and Birks 2004; Huntington et al. 2020; Moore 2011); however, some species may survive in locations that are characterized by local favorable environmental conditions outside their main area of distribution. Such locations are referred to as microrefugia (Ashcroft and Gollan 2013; Gubler et al. 2018; Rull 2009). Numerous studies analyzing different temporal and spatial microhabitat characteristics have indicated that the heterogeneity of habitats (Gallé et al. 2019), including their microclimates (Bramer et al. 2018), is underestimated. Free-air measurements obtained in standard meteorological stations at a height of 1.5–2.0 m over short grass or natural soil, in accordance with the guidelines of the World Meteorological Organization (WMO) (De Frenne et al. 2019; WMO 2018), provide general data about the macroclimate of an ecosystem. However, these measurements are not representative of a majority of the ecosystems. Although daily, seasonal, and interannual variations as well as multiyear trends converge with free-air measurements, the values of meteorological factors may significantly vary. The range of values of biotic and abiotic environmental factors determines whether or not a species exists in a given location. Therefore, due to the lack of monitoring and analysis of habitat properties, including microclimate, much information about individual species and their environmental requirements is unknown. To avoid extinction, an increase in global air temperature and the resulting habitat changes force species to track or adapt to the modified regional climate (Wasof et al. 2013). In recent years, species distribution models have been developed to aid in this analysis. Nevertheless, these models require specific measurement data for improvement and validation (Lembrechts et al. 2019).

Differences in macro- and microclimate may be especially large in ecosystems with high shading (forests), diverse landscape topography (mountains, young glacial landscapes) (Dobrowski 2011), and high soil moisture (e.g., wetlands) (Ashcroft and Gollan 2013; De Frenne et al. 2013; Słowińska 2016; Zellweger et al. 2019). This is also the case with urban areas, in which the phenomenon of urban heat island (UHI) is often observed (Manoli et al. 2020; Oke 1987; Stanley et al. 2019). In contrast to microclimate of forests, microclimate of peatlands has not been widely studied thus far (Chen et al. 1993; De Frenne et al. 2019; von Arx et al. 2012; Zellweger et al. 2019). For instance, De Frenne et al. (2019) performed a global analysis of the thermal buffering capacity of forests and showed that the mean and maximum understory temperatures were, on average, 1.7 ± 0.3 °C and 4.1 ± 0.5 °C cooler, respectively (mean ± s.e.m.), while the minimum understory temperature was 1.1 ± 0.2 °C warmer compared to the macroclimate outside the forest. A significant amount of climatological studies have also been conducted in cities. UHI, a common phenomenon in which temperatures in urban areas are higher than in surrounding rural areas, can be distinguished by a several Kelvin degrees warmer air temperature than the rural areas. For example, in Warsaw (Poland), the yearly average UHI index in 2011–2012 was a little more than 2.0 °C and the maximum index values were above 8.0 °C (Kuchcik et al. 2014; Zhao et al. 2014).

Peatlands are ecosystems with diverse water and nutrient supply, ranging from oligotrophic bogs to extremely rich fens (Charman 2002; Hajek et al. 2006; Succow 1988). Due to their geology, microtopography, water level, and nutrient inflow, they are also heterogeneous within a given type. All these factors have a significant impact on the composition of vegetation in peatlands (Chronakova et al. 2019; Rydin and Jeglum 2013). Most peatlands are extremely sensitive to interannual changes in meteorological conditions because their functioning is dependent on the precipitation-to-evapotranspiration ratio (Baldocchi et al. 2018; Samson et al. 2018; Yu et al. 2011). Studies that have been conducted so far on microclimate of peatlands were short term or a part of other environmental analyses, such as those focusing on greenhouse gas fluxes (Juszczak et al. 2012; Kellner 2001) or plant and microbial composition (Robroek et al. 2014). Worrall et al. (2019) conducted one of the few studies that analyzed the thermal properties of the surface of a reclaimed peatland. The authors demonstrated that a lowland peatland with a high water level could act as a cool, humid island during daytime when the agricultural area was considered on a landscape scale. Liao et al. (2013) reported similar findings based on a comparison of the microclimatic edge effects between wetlands and farmlands in northeastern China.

The object of the present study is the Linje mire, a microrefugium. Dwarf birch (*Betula nana*), which is a glacial relict, has been documented in a peat core since the period of Younger Dryas around 12,000 years ago (Noryśkiewicz 2005). Considering the knowledge gap in the long-term research on peatland microclimate, in this study we aimed to (i) analyze the local climate features of the mid-forest Linje mire, as an example of this type of ecosystem, in comparison to an open site, and determine the seasonal climate differences between the mid-forest mire and the open site, and (ii) examine the differences in microclimates of the microsites on the mire selected based on the diversity of plant communities, shading, and depths of the water table. We chose the peatland with one of the longest climate records in Poland for this study.

## Materials and methods

### Study site

**Fig. 1:**
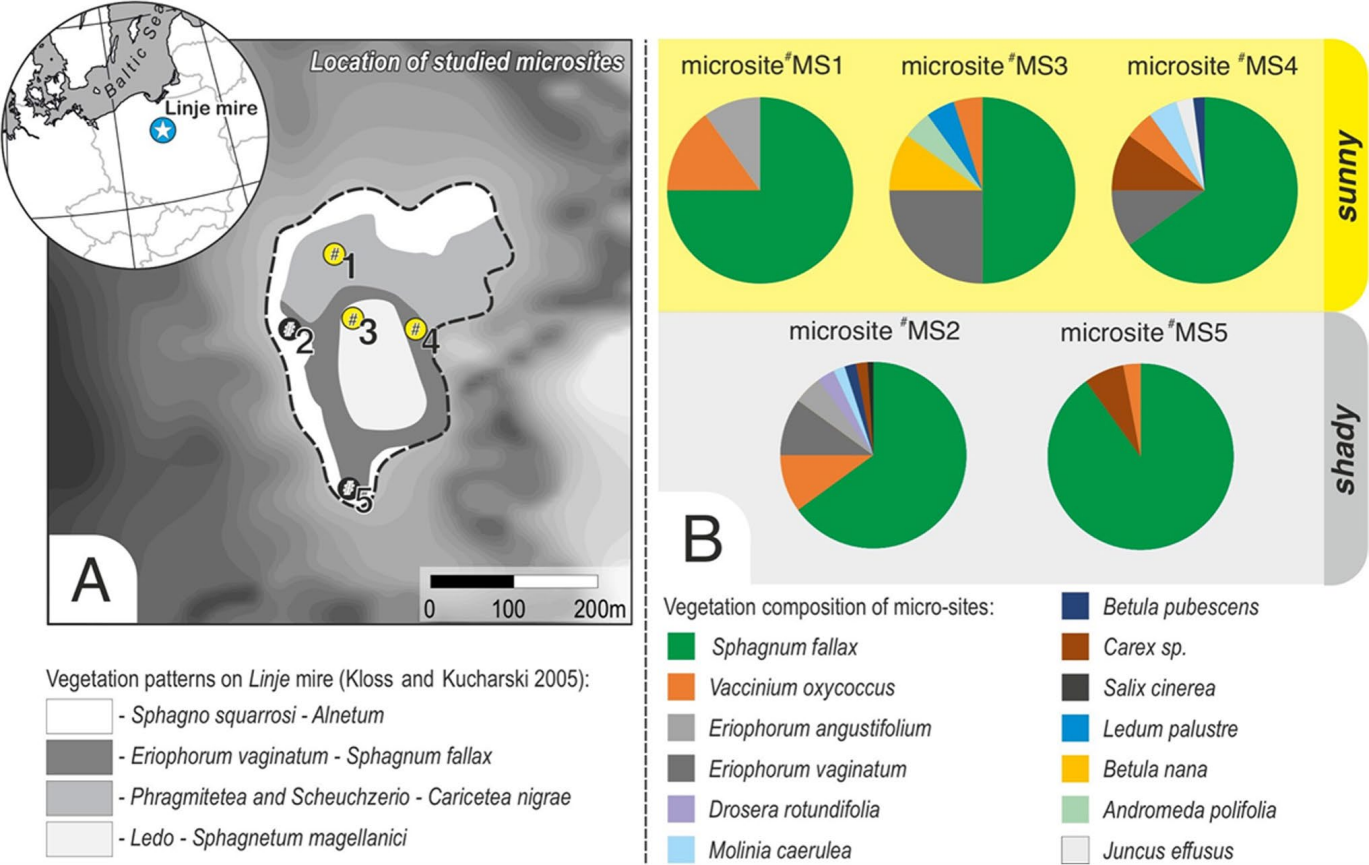
A Location of the Linje mire and the studied microsites (MS1–MS5), and B the vegetation composition of each microsite

The site chosen for this study was the mid-forest Linje mire, located in northern Poland (53° 11,015″ N, 18° 18,034″ E; Fig. 1). The mire is characterized by a temperate climate, which is influenced by both humid air masses from the Atlantic Ocean and dry inland masses. Its mean annual air temperature is 8.2 °C, and precipitation was recorded at 528.4 mm during the years 1951–2015 (station index 12250 located about 25 km from the mire, data from the Institute of Meteorology and Water Management). The mire is a poor fen with ombrotrophic vegetation that covers approximately 6 ha. It is situated in a terrain depression between a moraine hill and a sandur with a dunes system which is reflected by its water conditions (Słowińska et al. 2010). The averaged terrain denivelation in the mire’s direct catchment area reaches several meters. The hydrochemistry of peatlands, and thus vegetation, is significantly influenced by their geological surroundings (Wheeler and Proctor 2000). The Linje mire was drained in the second half of the nineteenth century by allowing water from the mire to flow out to the south (Słowińska et al. 2010). Drainage was stopped later, and the peatland is currently protected, but drainage ditches can still be seen on the surface (Lamentowicz et al. 2016). This study analyzed the microclimatic features of five microsites consisting of different plant communities as follows: MS1: *Phragmitetea* and *Scheuchzerio–Caricetea nigrae* (lawn structure comprising herb and moss layers); MS2 and MS5: *Sphagno squarrosi–Alnetum* (hummock–hollow structure comprising moss, herb, and low shrub layers); MS3: *Ledo–Sphagnetum magellanici* (hummock–hollow structure comprising moss and low shrub layers); and MS4: *Eriophorum vaginatum–Sphagnum fallax* (hummock–hollow structure comprising moss, herb, and low shrub layers) (Matuszkiewicz 2001). The vegetation composition of each studied microsite is described in Fig. 1. Microsites MS2 and MS5 were located close to the edge of the mire, and were therefore additionally shaded by tall *Salix cinerea* (MS2), *Betula pubescens* (MS2), and *Alnus glutinosa* (MS2, MS5).

### Field infrastructure and meteorological measurements

In order to determine the local climate features of the mire, the air temperature and humidity were recorded in its middle part (next to MS3; Fig. 1), and in the open site (reference) located 650 m away from the mire in a nearby village. The open site was situated on a lawn within the premises of a local company (Supplementary Information, Fig. 1). Temperature and relative humidity data loggers (HOBO U23 Pro v2; Onset Computer Corporation, USA) were installed at a height of 30 and 150 cm, and worked in 10-min intervals. The same measurements were taken at the reference site as well. Air temperature and humidity monitoring has been carried out in the mire and reference site from the year 2009, but for this study, we only considered the data for the period from April 2, 2012, to December 17, 2015, when all four data loggers (two at the mire and two at the reference site) were functioning correctly, and no statistical methods were needed to fill in data gaps.

As mentioned above, microclimatic studies were carried out in five microsites characterized by different plant communities and water conditions (Fig. 1). A data logger (EM-50; METER Group, USA), consisting of air temperature and humidity sensors placed in the radiation shield, photosynthetically active radiation (PAR) sensor, and leaf wetness (LW) sensor, was installed in each microsite. The air temperature, humidity, and PAR sensors were placed at a height of 30 cm, while the LW sensor was placed right above the surface of mosses. Groundwater wells with HOBO Water Level Data Loggers (Onset Computer Corporation, USA) were installed next to each micrometeorological station. Meteorological parameters were recorded in 10-min intervals, and the data were collected from April 15 to November 9, 2012, with the exception of the period from June 14 to July 8 when the logger failed at one of the plots. The depths of the water table were measured at 1-h intervals, and the results were averaged for the same period as meteorological parameters.

### Statistical analysis of meteorological data

The differences in the daily average, minimum and maximum temperatures, and vapor pressure deficit (VPD) between the mire and open (reference) site were used to determine the local climate features of the mire. VPD was calculated as the difference between the saturated and actual vapor pressure of air at each time step (10 min), and the results were averaged as other microclimatic variables (Allen et al. 1998). To determine the amount of heat in the ecosystem, growing degree days (GDD) with base 0° were estimated as a sum of all positive daily averages in degree Celsius for every year and their average value was calculated. The days of ground frost were calculated as days when the minimum air temperature at the height of 30 cm dropped below 0 °C while a positive maximum temperature occurred on a given day.

The microclimatic conditions of each microsite were characterized by determining the following indicators related to air temperature (*T*), relative humidity (*RH*), and VPD: averaged daily mean (*T*_mean_, *RH*_mean_, VPD_mean_), minimum (*T*_min_, *RH*_min_, VPD_min_) and maximum values (*T*_max_, *RH*_max_, VPD_max_), and absolute minimum and maximum values (only for temperature: *T*_minAbs_, *T*_maxAbs_). The diurnal temperature range (DTR) was calculated as the difference between the daily maximum and minimum values. The sum of PAR was calculated for the investigated period to compare the amount of solar energy reaching the ground at each microsite. GDD with base 0° were calculated as described above. Data obtained from the LW sensors were used to estimate the sum of hours with the presence of free water resulting from dew, fog, or rainfall close to the surface of mosses. This information indicates the microsite where the surface of mosses was most often wet. The course of temperature and VPD in July 2012 was also analyzed, based on hourly averages, to identify the differences in microclimates between the studied microsites during extremely hot days. Data on water table depths (WTDs) were used to determine the water conditions at each microsite. The groundwater level was recorded at 1-h intervals, and the results were averaged to the daily value and then to the investigated period.

The distribution of the differences was illustrated in box-plot charts, and the significance of differences was analyzed using the Kruskal–Wallis test. Calculations were made for the seasons (spring, summer, autumn, and winter), growing season, and year to determine the local climate features of the mire. Microclimates were characterized based on the entire investigation period. Statistical analyses were carried out in R program using “stats” (R Core Team 2020) and “ggplot2” (Wickham 2016) packages. The results were visualized using the Grapher application (Golden Software, LLC).

## Results

### Differences in air temperature and humidity between the mire and the open site

**Fig. 2:**
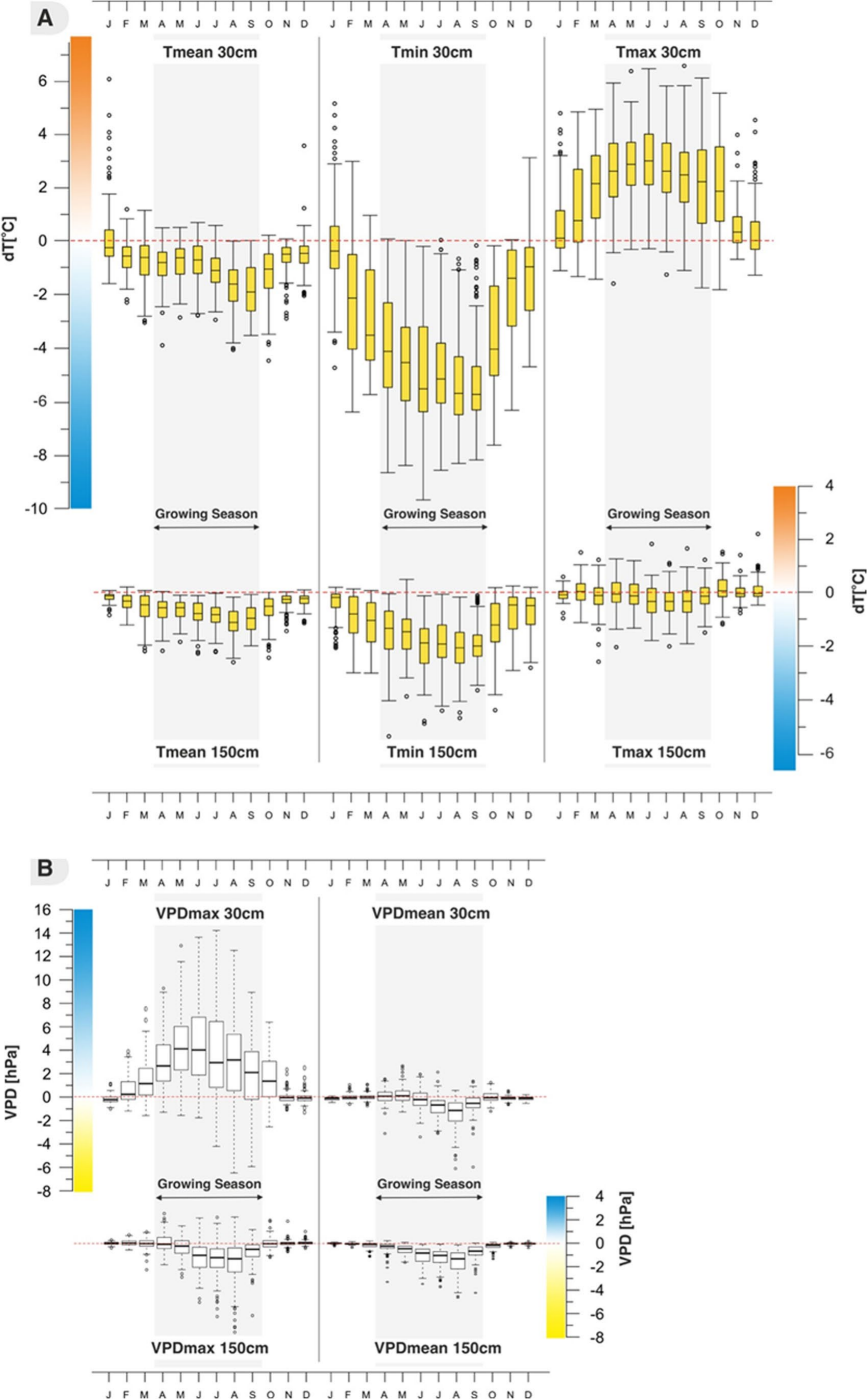
Monthly differences in A daily air temperature (mean, minimum, and maximum) and B VPD (mean and maximum) at a height of 30 and 150 cm between the mire and the open (reference) site for the period from April 2, 2012, to December 17, 2015

The years 2012–2015 were found to be warmer than the average of 1967–2015 (Bartczak et al. 2019). We observed a significant microclimatic separation between the mire and the reference site in the open area. Although the daily course of air temperature and humidity in the mire and the open site showed the same trends, the values were different. The mire was significantly cooler than the open site due to a higher decline in nighttime air temperatures. The differences in the air temperatures were more pronounced near the ground (30 cm) compared to that at the height of 150 cm, and were influenced by seasonal patterns and weather conditions. Among the temperature parameters, the largest differences were found in the values of minimal temperature (*T*_min_). The median difference in *T*_min_ was 3.9 °C near the ground and 1.4 °C at the height of 150 cm for a year, with the maximum temperature difference in summer reaching 9.8 °C in June (Fig. 2A, Table 1). In winter, the values of *T*_min_ were mostly higher in the mire than in the reference site, owing to snow cover. The mean air temperature at the mire was also lower by 0.81 °C near the ground and by 0.54 °C at the height of 150 cm for a year. In summer, the mire was cooler by 1.18 °C at 30 cm and by 0.92 °C at 150 cm, which was typical of *T*_mean_. The maximum temperature of the mire recorded near the ground was higher by 1.9 °C, while no significant difference was noted in the temperature recorded at the height of 150 cm (Table 1).

**Table 1:** Seasonal and yearly averages of differences in near-ground air temperature and VPD—mean, minimal, maximal, as well as DTR between the Linje mire and the open site for the period from April 2, 2012, to December 17, 2015

Microclimate variable (height)	Year (*n* = 1327)		Growing season (*n* = 702)		Spring (*n* = 336)		Summer (*n* = 338)		Autumn (*n* = 364)		Winter (*n* = 289)	
30 cm	Mean	Median	Mean	Median	Mean	Median	Mean	Median	Mean	Median	Mean	Median
*T*_mean_	− 0.97 ± 0.03	− 0.81*	− 1.23 ± 0.03	− 1.11**	− 0.84 ± 0.04	− 0.70	− 1.26 ± 0.05	− 1.18**	− 1.29 ± 0.05	− 1.00**	− 0.37 ± 0.06	− 0.46
*T*_min_	− 3.57 ± 0.07	− 3.90**	− 4.84 ± 0.03	− 5.28**	− 3.91 ± 0.11	− 4.10**	− 5.07 ± 0.11	− 5.51**	− 3.63 ± 0.12	− 3.80**	− 1.29 ± 0.12	− 0.90**
*T*_max_	1.90 ± 0.05	1.91**	2.59 ± 0.03	2.67**	2.61 ± 0.08	2.70**	2.60 ± 0.08	2.65**	1.53 ± 0.09	1.15*	0.72 ± 0.08	0.18
DTR	5.47 ± 0.10	5.83**	7.43 ± 0.03	7.60**	6.51 ± 0.16	6.85**	7.67 ± 0.15	7.93**	5.15 ± 0.18	5.00**	2.05 ± 0.16	1.20**
VPD_mean_	− 0.32 ± 0.07	− 0.19*	− 0.51 ± 0.04	− 0.40	0.03 ± 1.35	0.00	− 0.89 ± 0.06	− 0.71*	− 0.27 ± 0.03	− 0.20*	− 0.14 ± 0.01	− 0.10**
VPD_max_	2.05 ± 0.07	1.18*	3.36 ± 0.13	3.20**	3.06 ± 0.15	2.70**	3.63 ± 0.20	3.41**	1.22 ± 0.12	0.30	0.05 ± 0.05	− 0.20**
150 cm	Mean	Median	Mean	Median	Mean	Median	Mean	Median	Mean	Median	Mean	Median
*T*_mean_	− 0.66 ± 0.01	− 0.54*	− 0.87 ± 0.03	− 0.80*	− 0.67 ± 0.03	− 0.60	− 0.97 ± 0.03	− 0.92**	− 0.67 ± 0.03	− 0.50	− 0.30 ± 0.02	− 0.20
*T*_min_	− 1.40 ± 0.03	− 1.39**	− 1.83 ± 0.03	− 1.87**	− 1.46 ± 0.05	− 1.40**	− 1.99 ± 0.05	− 2.06**	− 1.34 ± 0.05	− 1.40**	− 0.72 ± 0.08	− 0.40*
*T*_max_	− 0.12 ± 0.01	− 0.11	− 0.22 ± 0.03	− 0.22	− 0.10 ± 0.03	− 0.10	− 0.36 ± 0.03	− 0.35	− 0.02 ± 0.02	− 0.10	− 0.01 ± 0.02	− 0.10
DTR	1.28 ± 0.10	1.21**	1.60 ± 0.04	1.60**	1.35 ± 0.06	1.20**	1.63 ± 0.06	1.66**	1.32 ± 0.05	1.35*	0.71 ± 0.05	0.40*
VPD_mean_	− 0.54 ± 0.02	− 0.26*	− 0.91 ± 0.03	− 0.70*	− 0.39 ± 0.02	− 0.30	− 1.29 ± 0.05	− 1.06**	− 0.27 ± 0.03	− 0.20	− 0.06 ± 0.01	0.00
VPD_max_	− 0.49 ± 0.03	− 0.15	− 0.89 ± 0.05	− 0.40	− 0.39 ± 0.02	− 0.30	− 1.44 ± 0.09	− 1.23	1.22 ± 0.12	0.30	− 0.02 ± 0.01	0.00

^**^Significance: < 0.001; ^*^significance: < 0.05

The mean VPD value (VPD_mean_) was lower at the mire compared to the open site in averaged differences. The average annual difference of VPD_mean_ was 0.32 hPa at the height of 30 cm and 0.54 hPa at 150 cm, and the largest differences occurred during the summer season, with 0.89 hPa at 30 cm and 1.29 hPa at 150 cm (Fig. 2B). The maximum VPD (VPD_max_), which occurred during the warmest time of a day, showed variation in differences. At the height of 150 cm, VPD_max_ was lower at the mire compared to the open site, but at 30 cm the value was higher. The average annual difference of VPD_max_ at 30 cm was 2.05 hPa, and 3.36 hPa in the growing season, while in July the difference reached almost 15.0 hPa (Fig. 2B). At 150 cm, VPD_max_ was lower by 0.49 hPa averaged in a year and 1.44 hPa in summer.

**Fig. 3:**
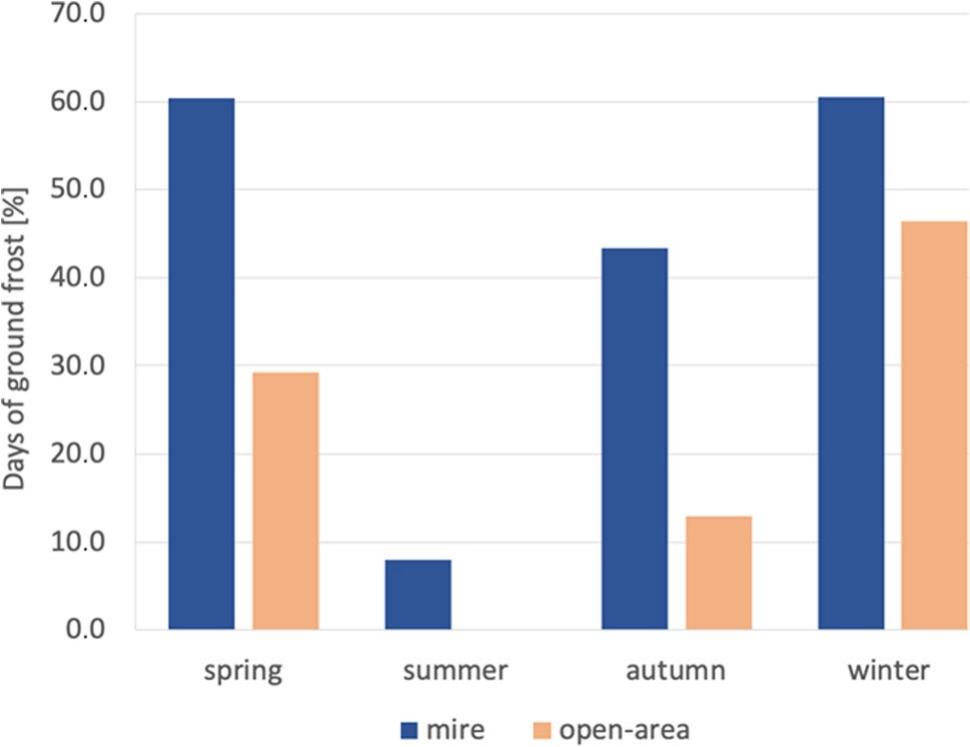
Days of ground frost in seasons and years at the Linje mire and the open site for the period from April 2, 2012, to December 17, 2015

At the mire, ground frosts (*T*_min_ < 0.0 °C; *T*_max_ > 0.0 °C) at the height of 30 cm were recorded for 42.4% days, while at the open site they were recorded for 21.0% days of the year (Fig. 3). Ground frosts were observed in every season, even in summer, at the mire and averaged for 8.0% days, whereas at the open site they were not recorded at all. Thermal resources defined based on the GDD (*T*_base_ = 0 °C) index were lower by 7.0% at the height of 30 cm (range 7–10%) and by 8% at 150 cm (range 5–9%) at the mire (average = 3244 GDD30cm and 3247 GDD150cm, respectively) compared to the open site.

### Diversity of microclimates at the Linje mire

A significant difference in microclimates was observed between the studied microsites (MS1–MS5). We found the highest sum of incoming PAR for MS1, which was identified as a site where incoming PAR completely reached the ground. Based on this, the relative reduction of incoming solar energy to the ground was calculated in the other microsites (18% for MS4, 24% for MS3, 49% for MS2, and 73% for MS5). The differences in the shading of microsites were reflected by other elements of microclimate. The highest daily mean temperature was observed for the most open site MS1 and for slightly shaded sites MS3 and MS4 (Table 2). The value of *T*_mean_ was the lowest in the shadiest sites MS2 and MS5, but the differences between the sites were statistically insignificant. Extreme air temperatures, both minimum (*T*_min_) and maximum (*T*_max_), showed highly significant differences. Average and absolute *T*_min_ were twice as low at the most open sites (MS1, MS3, and MS4), where the average values ranged from 2.4 to 3.0 °C, while in the shaded sites (MS2 and MS5) the values reached 4.3–6.4 °C. The highest maximum temperature was noted in the partly shaded sites (MS3 and MS4), where the absolute values were 39.5–40.0 °C, while in the most shaded sites (MS2 and MS5) the maximum temperature was 33.7–33.8 °C. DTR was found to be the smallest for the sites MS2 and MS5. Heat accumulation in microhabitats was determined by GDD (*T*_base_ = 0 °C), based on which MS4 was identified as the warmest and MS2 as the coldest (Table 2).

**Table 2:** Microclimatic characteristics of microsites during the period from April 15 to November 9, 2012 (except June 14–July 8)

Microclimate variables		Microsites				
		**MS1**	**MS2**	**MS3**	**MS4**	**MS5**
PAR [MJ]	sum^**^	1095.7	554.6	838.1	897.2	294.2
	mean	12.6 ± 0.4	11.6 ± 0.4	12.5 ± 0.4	12.3 ± 0.4	12.2 ± 0.4
*T*_air_ [°C]	min_avg_^**^	2.4 ± 0.4	4.3 ± 0.4	2.5 ± 0.4	3.0 ± 0.4	6.4 ± 0.3
	min_abs_	− 12.6	− 8.2	− 11.3	− 11.0	− 6.2
	max_avg_^**^	22.3 ± 0.5	19.3 ± 0.5	23.2 ± 0.6	23.7 ± 0.6	19.0 ± 0.5
	max_abs_	38.0	33.7	39.5	40.0	33.8
	DR_avg_	19.8 ± 0.5	15.1 ± 0.4	20.7 ± 0.5	20.7 ± 0.5	12.7 ± 0.4
GDD [*T*_base_ = 0°]	sum	2276	2173	2359	2456	2340
	mean**	78.5 ± 0.7	86.6 ± 0.7	79.6 ± 0.7	80.3 ± 0.6	85.5 ± 0.7
RH [%]	min_avg_**	50.6 ± 1.1	67.5 ± 1.4	50.6 ± 1.3	49.7 ± 1.3	65.3 ± 1.3
	max_avg_**	96.1 ± 0.1	97.1 ± 0.1	96.9 ± 0.1	96.2 ± 0.1	96.6 ± 0.1
	DR_avg_**	45.5 ± 1.1	29.6 ± 1.3	46.3 ± 1.3	46.6 ± 1.3	31.3 ± 1.2
	mean**	5.1 ± 0.3	2.8 ± 0.2	5.1 ± 0.3	4.9 ± 0.3	2.9 ± 0.2
VPD [hPa]	min_avg_**	0.4 ± 0.0	0.3 ± 0.0	0.3 ± 0.0	0.4 ± 0.0	0.4 ± 0.0
	max_avg_**	15.4 ± 0.7	9.2 ± 0.6	17.0 ± 0.9	18.0 ± 0.9	9.4 ± 0.6
	DR_avg_**	15.1 ± 0.7	8.9 ± 0.6	16.7 ± 0.9	17.6 ± 0.9	8.9 ± 0.5
LW [h]	sum^**^	2169	3327	2527	2055	2636
WTD [cm]	mean	11.9	9.1	18.9	20.6	6.0

^**^Significance: < 0.001; ^*^significance: < 0.05

**Fig. 4:**
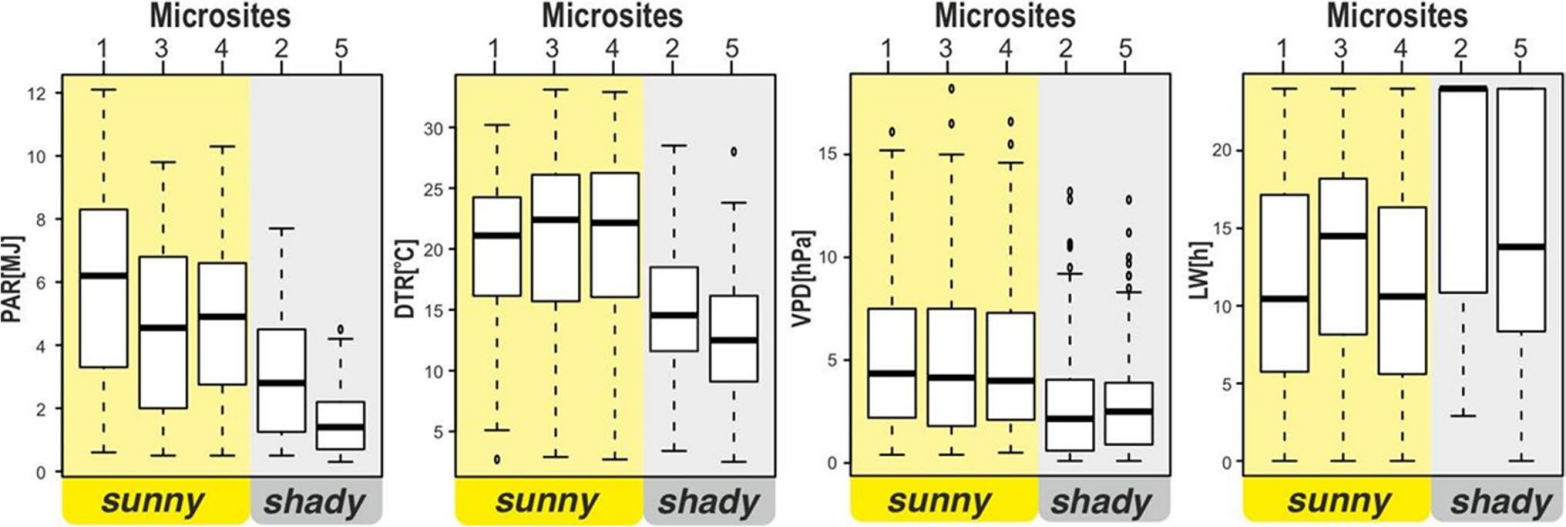
Daily sum of PAR, DTR, VPD, and LW for the period from April 15 to November 9, 2012 (except June 14 to July 8)

Analysis of relative humidity and VPD indicated that MS2 and MS5 sites had the highest humidity. The average daily relative humidity (RH_mean_) was 85.5–86.6% and average VPD (VPD_mean_) was 2.8–2.9 hPa. The LW duration was the longest in MS2 (3327 h), while in MS5 it was 2636 h for the investigated period. The driest places were MS1 and MS3, where the daily average relative humidity was 78.5–79.6% and VPD was 5.1 hPa. More importantly, the 95th percentile of the daily average VPD was 15.0 hPa in MS1, MS3, and MS4 and only 7.1–8.0 hPa in MS2 and MS5 (Fig. 4). The LW duration was the shortest in MS4 (2055 h). The microsites MS2 and MS5 were also found to be the wettest based on the values of WTD. The average groundwater level in these sites was 9.1 and 6.0 cm, respectively. MS4 located by the dune was identified as the driest site with an average WTD of 20.6 cm.

**Fig. 5:**
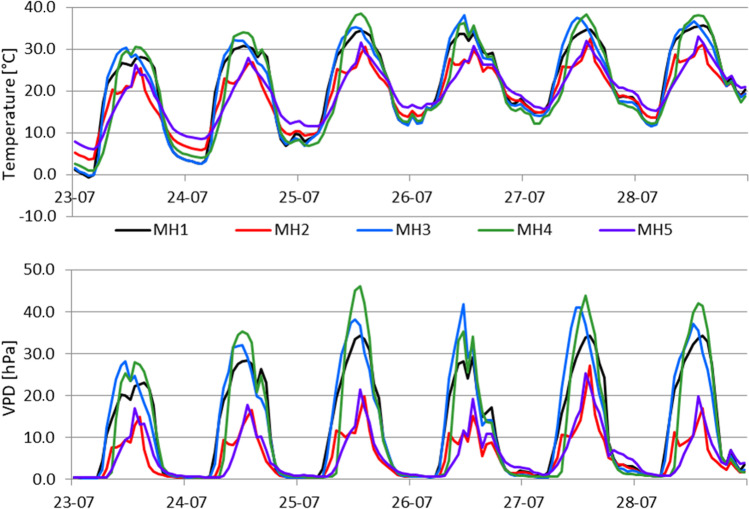
Daily time course of air temperature and VPD at the microsites MS1–MS5, during July 23–28, 2012

The differences in microclimate between the studied microsites were especially pronounced on hot and dry days, as occurred from July 23 to 28, 2012. The daily time courses of air temperature and VPD recorded at each microsite are plotted in Fig. 5. The differences in maximum daytime air temperatures between the most shaded (MS2 and MS5) and more open microsites (MS1, MS3, and MS4) ranged from 5.1 to 8.2 °C (an average difference of 6.9 °C). In the open microsites, greater heat emission resulted in a higher decline in air temperature during the night and early morning hours, and on July 23, ground frosts occurred at MS1 and MS3, while *T*_min_ at MS5 was 6.0 °C. Maximal VPD was found to be much higher in the open microsites (MS1, MS3, and MS4) reaching a value of 46.1 hPa (MH4), while in the shaded microsites (MS2 and MS5) it was approximately 20.0 hPa.

## Discussion

### Climatic distinctiveness of the mire

According to Dobrowski (2010), refugia are characterized by climates that are decoupled from regional averages. In this study, we found that the mire was significantly cooler than the reference site. Even though the differences in mean annual temperatures between the mire and the reference site were relatively small (less than 1 °C; Table 1), they resulted from large differences in the minimum daily air temperature, especially during the growing season which is the most important for living organisms. The *T*_min_ and *T*_max_ values of the reference site and the mire differed by several degrees. Other studies conducted in raised bogs revealed that they differ from the surrounding landscape in both lower and higher temperature extremes, and were characterized by a higher chance of ground frost in summer nights and a lower mean temperature (Eggelsman 1980; Ermich 1972; Peus 1932).

The isothermal map of the mean annual air temperature of Poland shows that a temperature difference of 1 °C reflects a spatial shift to the north or east by about several dozen kilometers toward a cooler climate (Climate Maps of Poland, Institute of Meteorology and Water Management, NRI; https://klimat.imgw.pl/pl/climate-maps). Thus, geographically speaking, the local climate of the mire corresponds to the regional average for cooler areas.

It was observed that the local climate and microclimates in the mire were influenced by the synergy of factors, including topography, wetness conditions, and vegetation composition. The location of the mire in a small depression, its relatively small area (about 6.0 ha), and the surrounding forest favor the formation of a cold air pool (Geiger et al. 1995; Lundquist 2008), where cooler and heavier air is held closer to the ground, with warmer air above.

Many peatlands in Central Europe are at a similar location (Tobolski 2000), and thus may be characterized by similar local climate features.

The composition and properties of vegetation, as well as the hydrological conditions which are dependent on the annual sum and distribution of precipitation, are undeniably one of the most important factors influencing microclimate. Postglacial peatlands in Central Europe are mainly dominated by *Sphagnum* moss. A several-meter layer of such sediments can be found in some of these peatlands, as in our study site. However, an acrotelm, which is the active layer, responds to meteorological changes through changing groundwater levels (Morris et al. 2011; Tobolski 2000). Apart from plant composition, which is influenced by a variety of factors, water level and surface wetness determine the thermal properties of *Sphagnum* mosses (Ellenberg 1988).

Due to the extremely low heat capacity and thermal conductivity of dry *Sphagnum*, heat is transferred into and out of the dry peat surface at a slower rate than wet peat (Loranty et al. 2018; Petrone et al. 2004). Furthermore, dry peat mosses have a poor capillary uptake capacity and cannot draw water from deeper layers (Romanov 1968). Thus, the surface layer formed by *Sphagnum* mosses acts as an insulating layer filled with air, contributing to an increase in albedo (Price 1996). It protects the deeper layers from water loss and heating up, thereby preventing heat transfer from these layers to the surface during cooling at night. The Linje peatland was drained at the end of the nineteenth century (Słowińska et al. 2010). The remains of ditches still slightly increase water outflow from the peatland, increasing the interannual and seasonal hydrological instability. Even short periods of no rainfall in summer cause a significant decrease in groundwater level, resulting in drying of the surface layer (Samson et al. 2018; Słowińska et al. 2010; Słowińska 2016). Therefore, especially in summer, air temperature in the mire fluctuated more than at the reference station. The dry surface of *Sphagnum* moss strongly heated during the day, which led to higher daily maximum air temperatures, and the surface cooled down at night, resulting in significantly lower daily minimum air temperatures. The higher air temperature was responsible for the higher air VPD, which in turn determines the moss evaporation rate (Heijmans et al. 2004). Heterogeneous vegetation structure influences the microtopography of the mire which has a cascading effect on physical parameters as well as microbiology (Fournier et al. 2020; Malhotra et al. 2016; Rydin and Jeglum 2013).

### Microclimatic differences on the peatland—effect of shading, vegetation, and wetness

The microsites analyzed in our study represented four different types of vegetation. Two of the studied microsites (MS2 and MS5) were shaded from direct sunlight by trees with a high groundwater level. The trees and the south and west edge of the mire, which was right next to the forest line, obscured the horizon, thus directly influencing the light conditions compared to the other more open microsites (MS2, MS3, and MS4). The incoming solar energy was therefore limited in MS2 and MS5. The reduced sky view factor of the sites beneath the canopy reduced long-wave radiative losses, and as a result, the daily air temperature ranges of the shaded sites were smaller than those in the open sites. Additionally, the shaded sites had a high groundwater level, with wet mosses having greater heat capacity and thermal conductivity than dry ones, and therefore, the diurnal air temperature fluctuations were lower. At the open microsites, higher solar radiation contributed to higher air temperature and thus lower air humidity during the daytime. At night, temperature and humidity were higher in the shaded stands. Wet and shaded sites having a high groundwater level had lower amplitudes of daily air temperature, owing to the lower maximum and higher minimum temperatures. In general, the studied sites were cooler during the investigation period. The data obtained from the LW sensors indicated that the shaded stands were also wetter in terms of air humidity and due to the presence of dew deposits. At open microsites, VPD was almost twice as high, potentially causing greater ecological stress for microorganisms and plants (Jassey et al. 2018; Pappas et al. 2020). Moreover, the maximum air temperature in these microsites was 4 °C higher compared to the shaded sites. Our findings show that the differences in microclimate between the sites located close to each other may significantly vary, and based on a study by Heijmans et al. (2004), it can be concluded that microclimate is the primary factor determining moss evaporation rates.

According to climate change projections, the median of climatic water balance (difference in precipitation and evaporation) will decrease from − 32 mm (1971–2000) to − 50 mm (2061–2090) (Szwed et al., 2010). In the case of peatlands fed by rain-derived water (ombrogenic ones) and shallow groundwater (topogenic ones), the projected climate change will cause a water-level drawdown and the entry of shrubs and trees due to succession (Laiho et al., 2003). This will change a number of relationships, including microclimatic conditions, of both ground and near-ground layer (Heijmans et al. 2013; Laiho et al. 2003). Our study showed that the shaded microsites had a completely different microclimate. However, it is difficult to determine which factor—shade or high groundwater level—was more important in keeping them cool.

### Peatlands as climate refugia

The specific local climate of peatlands is determined by various factors—including topography, water conditions, vegetation composition, and specific climatic conditions associated with them (Eggelsman 1980; Ermich 1972; Peus 1932). Due to their unique features, peatlands often act as refugia for glacial relicts such as *B. nana* or *Rubus chamaemorus* as well as insects such as *Somatochlora alpestris* (Głowaciński and Nowacki 2004; Zarzycki and Mirek 2006). The species *B. nana* has been documented in the peat core at our site since the period of Younger Dryas around 12 thousand years ago (Noryśkiewicz 2005). However, a more in-depth investigation is required to determine the factors enabling the survival of this species.

Human activities, such as afforestation and peat extraction, disrupt the natural functioning of peatlands (Łuców et al. 2020; Lamentowicz et al. 2019b). Disturbed peatlands are hydrologically unstable and respond more quickly to normal seasonal and interannual changes in meteorological conditions. Moreover, higher climate fluctuations and frequent extreme events, including droughts, will undoubtedly have a negative impact on the functioning of peatlands (Gallego-Sala et al. 2018; Loisel et al. 2021; Swindles et al. 2019). This can result in peatlands overgrowing, and necessitating active management for raising the groundwater level or removing trees to keep them relatively stable. Maintaining stable water conditions in peatlands in this climate zone, however, will be a major challenge. Swindles et al. (2019) showed that the paleohydrological record from the last 300 years indicates a decreasing trend of the groundwater table in European peatlands, which is attributed to climate warming and human activities. Long-term persistence of a low groundwater level results in tree encroachment (Heijmans et al. 2013), which drastically alters microclimates and other processes (Davis et al. 2019; Limpens et al. 2014; Stralberg et al. 2020; Winter 2000).

### Importance of microclimatological research for paleoecology

To interpret the findings of paleoecological research, it is important to study the ecology of peatlands as well as define their resilience to recent climate changes (Hapsari et al. 2018; Harris et al. 2020; Page and Baird 2016). For example, testate amoebae are one of the proxies used in reconstruction and bioindicators (Mitchell et al. 2007a, 2007b) and can be used to reconstruct past hydrological dynamics (Lamentowicz and Mitchell 2005; Lamentowicz et al. 2015). However, these organisms may respond to meteorological factors, including the amount of incoming solar radiation (Herbert et al. 2018; Jassey et al. 2015; Lamentowicz et al. 2020; Marcisz et al. 2014). Only through detailed ecological research on the microbial communities of peatland, including meteorological measurements in microscale, one can answer the question of how their functional traits vary in time and space (Marcisz et al. 2020).

In this study, paleoecological results showed that *B. nana* was present in the mire during the Late Glacial period and throughout the Holocene (Noryśkiewicz et al. 2005). Despite significant climatic changes during that time period, which directly as well as indirectly influenced the development and transformation of the mire from a rich fen (with a mix of *Sphagnum* and vascular plants) to a poor fen (dominated by *Sphagnum*), *B. nana* has survived to the present day (Marcisz et al., 2015). The results of our study showed that such transformation was possible through a synergy of individual terrain elements, and was mediated by physical refugia features (e.g., peatland located on the boundaries of two watersheds, steep ice melt-out forms) and ecosystem-protected mechanism (climatic buffering, light and hydrological competition) (Shur et al. 2007; Stralberg et al. 2020; Waddington et al. 2015).

A comprehensive understanding of long-term peatland monitoring will allow bridging the gap between ecology and paleoecology. This knowledge may help us better interpret the reconstructed data from the peat archive. By combining reconstruction, observation, and experimentation, we can go beyond and determine the tipping points for the ecosystems under study (Lamentowicz et al. 2016, 2019a; Seddon et al. 2014). Furthermore, the framework of monitoring and the results presented here can be readily extended to phenological or microbiological biodiversity research. A real data example was used to demonstrate our approach to define the characteristics and heterogeneity of peatland microclimates as well as the inertia/mitigation of climate changes.

## Conclusions

Our long-term measurements of meteorological parameters on the peatland provided a novel insight into the local climate features in the context of seasonality of this ecosystem. We investigated the unique climate features and microclimate differences in a mid-forest peatland located in northern Poland, in terms of shading due to shrubs and trees and wetness effects.

The studied peatland was cooler on average than the open reference site, due to higher drops in night air temperature. During the growing season, the surface layer of the peat often dried out, causing changes in the physical properties of mosses and greater fluctuations in diurnal air temperature at the ground layer. The site was a drained object that reacted quickly to changes in meteorological conditions, especially the amount and distribution of precipitation and temperature during growing seasons, both of which largely influence the evapotranspiration rate. The microclimatic conditions of the peatland were significantly affected by WTDs and shade. Wet and shaded sites were cooler than those with a lower water level and receiving a higher amount of solar radiation, and were also less exposed to extreme daily temperatures. This is important in the context of climate change, as an increase in global air temperature will lead to an increase in evapotranspiration rate, and subsequently a decrease in the water table in peatlands directly dependent on meteorological conditions. Overgrowth of trees is a natural effect that lowers the groundwater level, resulting in an increase in shading and a secondary impact on evapotranspiration in the sites. Our research is part of the discussion on the importance of microclimatic studies of ecosystems in climate change. Although the results presented here are specific to one mire, and were obtained from a case study, they can be applied to similar objects on earth in similar physiographic conditions. They can also be used to model species distribution.

The trajectory of the earth system is currently changing as a result of dramatic human-driven climate changes (Steffen et al. 2018). These climate changes have irreversible consequences for the ecosystems, society, and economy. Therefore, it is critical to understand under what conditions ecosystem function can be corrected by management strategies in the future. The findings presented in this paper make an important contribution to our understanding of the functioning of peatlands in time and space.

## Supplementary Information

Below is the link to the electronic supplementary material.

Supplementary file1 (JPG 331 kb)
