# Supplementary figures and images for: Long-term microclimate study of a peatland in Central Europe to understand microrefugia
Source: Int J Biometeorol. 2022 Feb 3;66(4):817–32. doi: 10.1007/s00484-022-02240-2 (PMC8948114; doi:10.1007/s00484-022-02240-2)

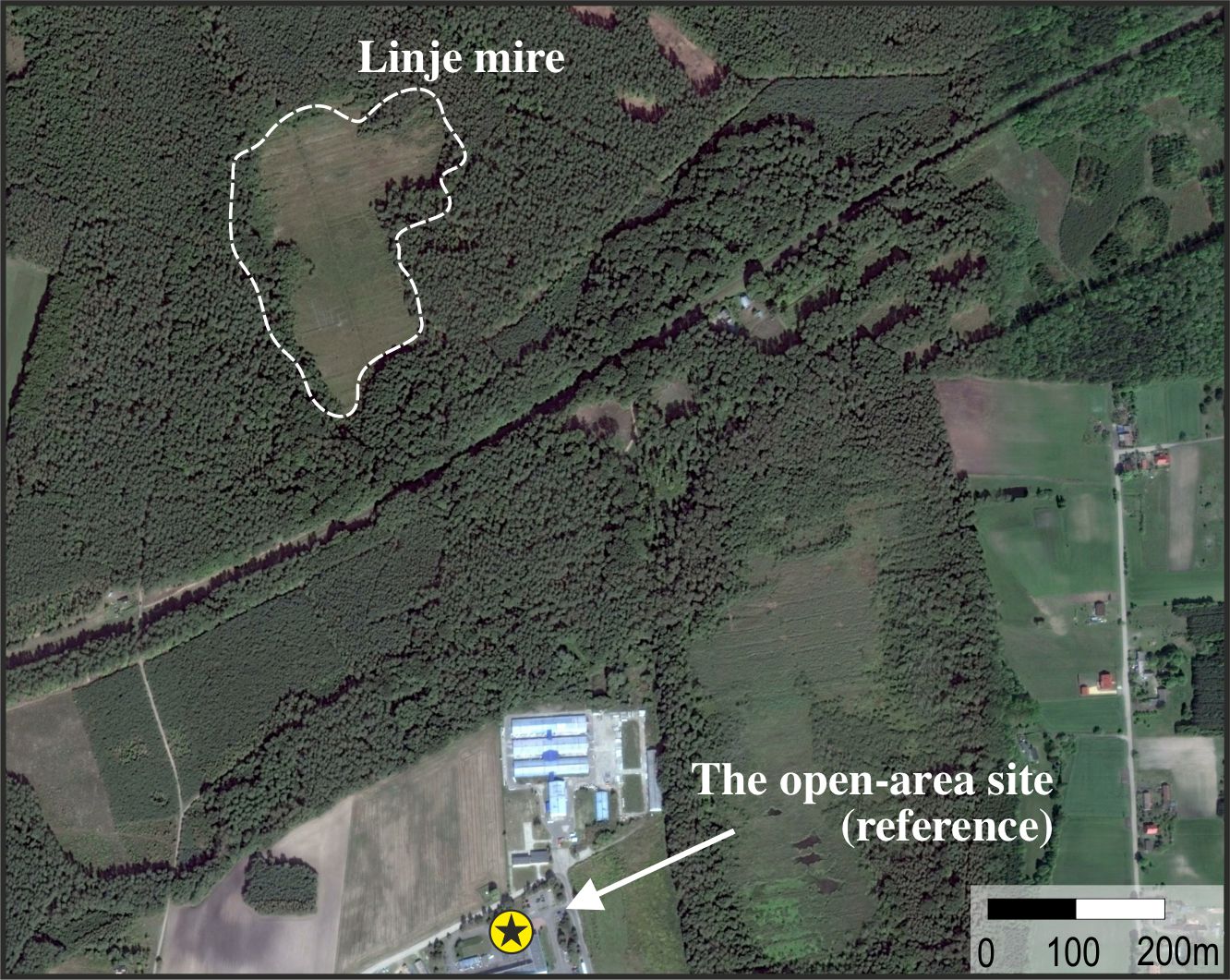

Supplement: Supplementary file 1 — Supplementary file1 (JPG 331 kb) [file 484_2022_2240_MOESM1_ESM.jpg]
